# Anthropometric and Micronutrient Status of School-Children in an Urban West Africa Setting: A Cross-Sectional Study in Dakar (Senegal)

**DOI:** 10.1371/journal.pone.0084328

**Published:** 2013-12-31

**Authors:** Marion Fiorentino, Guillaume Bastard, Malick Sembène, Sonia Fortin, Pierre Traissac, Edwige Landais, Christèle Icard-Vernière, Frank T. Wieringa, Jacques Berger

**Affiliations:** 1 Groupe de Recherche et d’Echanges Technologiques (GRET), Dakar, Senegal; 2 Division du Contrôle Médical Scolaire (DCMS), Ministère de l’Education Nationale, Dakar, Senegal; 3 Institut de Recherche pour le Développement (IRD), UMR 204 Nutripass, IRD-UM2-UM1, Montpellier, France; University of South Australia, Australia

## Abstract

**Background:**

Urban areas in West Africa are not immune to undernutrition with recent urbanization and high food prices being important factors. School children often have a poor nutritional status, potentially affecting their health and schooling performance. Yet, generally school children do not benefit from nutrition programs. The objective of the study was to assess the anthropometric and micronutrient status of children from state schools in the Dakar area.

**Methods:**

School children (n = 604) aged from 5 to 17 y (52.5% girls, 47.5% ≥10 y) were selected through a two-stage random cluster sample of children attending urban primary state schools in the Dakar area (30 schools × 20 children). The prevalence of stunting (height-for-age<−2 z-scores) and thinness (BMI-for-age<−2 z-scores, WHO 2006, and three grades of thinness corresponding to BMI of 18.5, 17.0 and 16.0 kg/m2 in adults) were calculated from weight and height. Hemoglobin, plasma concentrations of ferritin (FER), transferrin receptors (TfR), retinol binding protein (RBP), and zinc, and urinary iodine concentrations were measured. Correction factors were used for FER and RBP in subjects with inflammation determined with C-reactive protein and α1-acid-glycoprotein.

**Results:**

4.9% of children were stunted, 18.4% were thin, 5.6% had severe thinness (BMI-for-age<−3 z-scores). Only one child had a BMI-for-age>2 z-scores. Prevalence of anemia, iron deficiency and iron deficiency anemia was 14.4%, 39.1% and 10.6% respectively. 3.0% had vitamin A deficiency, 35.9% a marginal vitamin A status, and 25.9% zinc deficiency. Urinary iodine was <50 µg/L in 7.3% of children and ≥200 µg/L in 22.3%. The prevalence of marginal vitamin A, zinc deficiency, high TfR was significantly higher in boys than in girls (P<0.05). Height-for-age and retinol were significantly lower in participants ≥10 y and <10 y respectively.

**Conclusion:**

Undernutrition, especially thinness, iron and zinc deficiencies in school children in the Dakar area requires special targeted nutrition interventions.

## Introduction

The prevalence of food insecurity in Sub-Saharan Africa is the highest in the world, with rates as high as 30% of the population being undernourished [1]. For instance, 26% of the population in Senegal is undernourished, which ranks it 155^th^ over 187 countries in the 2011 Human Development Index [2]. Due to considerable rural migration and urbanization during the last decades, 42% of the Senegalese population now live in urban areas [3]. Many households in the Dakar area are therefore without basic infrastructures, while simultaneously vulnerable to food insecurity [4]. Moreover, albeit the general assumption that urban populations have access to more diversified foods, studies in West Africa haveshown that micronutrient status can be low in urban areas [5].

Nutrition interventions generally neglect school children despite their high prevalence of malnutrition and micronutrient deficiency [6]. Iodine, iron and folic acid micronutrient deficiencies affect the development of the brain and cognitive functions of school children [7]. Iodine deficiency, even mild, could impede full intellectual potential [8] with differences in intellect as large as 10–15% between iodine deficient and non-deficient populations. On the other hand, deficiencies of vitamin A and zinc are associated with different scenarios affecting school performance, such as absenteeism due to increased morbidity [9], [10].

Data on nutritional status of school children in Senegal are scare and recent data on school children living in urban areas are lacking. A study in 1994 conducted on a representative sample of 774 children (aged from 5 to 15 y) of state primary schools of the Dakar department showed that 34% of pupils were anemic, 10% underweight, 5% stunted and 11% wasted [11]. More recent, a study on Senegalese food practices and nutrition was conducted to identify strategies to reduce malnutrition [12]. New food practices are emerging in urban areas with food prepared at home decreasing, while street foods purchases increased. Furthermore, when a child reaches school age, it can be observed that mothers take less care of their children’s diet. The rarity of school canteens in urban state school further reduces meal opportunities for children [12]. While school children consume less meals with adults, an increasing number of households can’t afford to spend money on snacks for their children. Many children declare having difficulties to concentrate in class due to hunger [4].

It is therefore likely that the nutritional intake of foods from school-aged children in Senegalese urban areas is inadequate. The main objective of the study lies in the assessment of the anthropometric and micronutrient status of school children attending state primary schools in Dakar and suburbs. The Dakar region was selected because it has the highest population density (4513 people/km2) and schooling rate (90%) of the country [13].

## Participants and Methods

### Study Area and Population

The Dakar region, including the capital Dakar and its suburbs, is located on the Cap-Vert peninsula. It represents 0.3% of the Senegal area and 21% of its population. The study was conducted in February-March 2010. The target population, children from state primary schools of Dakar region, was estimated to be over 200,000 children, distributed among 370 state schools.

### Study Design

The study was a cross-sectional survey. A two-stage cluster sampling method was chosen with schools considered as primary sampling units. Within randomly selected schools, and without criteria for age and gender, children were randomized as final sampling units. The required number of participants was calculated following the formula n = (1.64^2^×P×(1**−**P))/m^2^; where the prevalence P was estimated equal to 0.5 and the expected precision m for this prevalence to be 0.05. Moreover, a design effect equal to 2 was chosen [14] leading to a required sampling size of 538 children. Thirty schools were randomly selected, with 20 children per school, giving a final sample size of 600 children. The regional education authority provided the list of schools, while children were randomly selected from lists provided by the school directors.

### Data Collection

Mothers or caretakers of children were surveyed at home for socioeconomic characteristics of the households. Birth dates of children were recorded from the school lists and checked with birth certificate or identity card. When official documents were missing, children’s mothers or caretakers were questioned using a local events calendar. Children were defined as participants less than 10 y, and teenagers as participants from 10 y and above, according to the World Health Organization (WHO) definition [15].

The nutritional assessment period lasted 6 weeks between February to March 2010, with one school visited each day. Blood, urine samples and anthropometric measurements were collected at schools in the morning between 8 and 10 AM. They were then verified for their identity. In order to define their fasting status, children were also asked the last time they consumed food. Weight and height were measured without footwear and wearing minimal clothes. To avoid between-measurers variability, all anthropometric measurements were performed by only one trained anthropometrist. The accuracy of the scale and the stadiometer was checked every day using a set of 2 calibration weights and one calibration tape. Height was measured twice to the nearest 0.1 cm on a Seca 214 stadiometer and mean values were used. When differences between two measures of height for the same child exceeded 0.5 cm, measurements were repeated. Weight was measured once to the nearest 100 g on a Pespe T125 Terraillon scale. Height-for-age (HAZ) and BMI-for-age z-scores (BAZ) were calculated according to the WHO 2006 reference [16]. Stunting was defined by HAZ<−2 z-scores. Overweight was defined by BAZ between 1 z-scores and 2 z-scores and obesity by BAZ >2 z-scores. Two growth international references were used to classify thinness: the WHO reference defining mild, moderate and severe thinness respectively by z-scores between [−2; −1 [, between [−3; −2 [and <−3 [16]; the IS reference defining grades 1, 2, 3 of thinness corresponding to WHO cut-off for BMI of 16, 17, and 18.5 respectively at age 18 [17].

Four (4) ml of venous blood were collected in a Terumo heparin Venosafe vacutainer with heparin by an experienced phlebotomist (using sterile single-use material). Urine was taken in a sterile container. Urine and blood samples were stored immediately in an icebox containing ice-packs and transported to the laboratory within a maximum of 3 hours after the first sample withdrawal.

Hemoglobin concentration (Hb) was measured at arrival at the laboratory of Pasteur Institute in Dakar on whole blood with HemoCue® Hb 201+ and HemoCue controls (Hemotrol low, medium, high, HemoCue®). Moreover 5% of blood samples were measured by hematology analyzer Cell-dyn® as an external control. Blood samples were centrifuged at 4000×g for 10 minutes at **−**4°C, plasma aliquoted in 4 eppendorf tubes and stored at **−**20°C for 6 weeks until completion of the field work and sent with dry ice to the Nutripass laboratory of the Institut de Recherche pour le Développement (IRD, Montpellier, France) for zinc and iodine measurements and to the CBS laboratory (Willstaett, Germany) for determination of retinol-binding protein (RBP), C-reactive protein (CRP), ferritin (FER), soluble transferrin receptor (TfR) and α1-acid-glycoprotein (AGP). RBP, FER, TfR, CRP, AGP were measured by a sandwich enzyme-linked immunosorbent assay (ELISA) technique [18]. Plasma zinc was measured by flame atomic absorption spectrophotometry (AAS), using trace-elements free procedures and urinary iodine (UIC) was measured using an ammonium persulfate method [19].

Inflammation was determined by elevated CRP (>5 mg/L) and/or elevated AGP (>1 g/L) allowing differentiation between incubation phase (high CRP), convalescence phase (both AGP and CRP elevated) and late convalescence phase (elevated AGP only) [20]. Anemia was defined by Hb below cut-offs depending on age and gender: 115 g/L for participants <12 y; 120 g/L for teenagers between 12 and 15 y and girls ≥15 y; 130 g/L for boys ≥15 y ); severe anemia was defined as Hb <70 g/L; depleted iron stores were defined by corrected FER <15 µg/L [21]. Correction factors of FER were 0.77, 0.53 and 0.75 for participants respectively in incubation, early convalescence, and late convalescence phases [20]. Iron tissue deficiency was defined by TfR >8.3 mg/L [18]. Low FER and high TfR are both considered as indicators of iron deficiency (ID) [21]so ID was defined by iron stores depleted (low FER) and/or iron tissue deficiency (high TfR). Body iron was calculated according to the formula of Cook [22] [22]: body iron (mg/kg) = **−**(log (TfR/FER ratio)**−**2.8229)/0.1207. “Body iron deficiency” was defined by body iron <0. Vitamin A status was measured by RBP concentration which reflects plasma retinol concentration because RBP occurs in a 1∶1:1 complex with retinol and transthyretin [23]. RBP concentrations were corrected in participants with inflammation by factors 1.15, 1.32, 1.12 respectively for incubation, early convalescence and late convalescence [24] [24]. Vitamin A deficiency (VAD) was defined by corrected RBP<0.7 µmol/L and ≥0.35 µmol/L and severe VAD was defined by corrected RBP<0.35 µmol/L [23]. Marginal VAD was defined for corrected RBP values ≥0.7 µmol/L and <1.05 µmol/L [25].

Zinc deficiency (ZnD) was defined by plasma zinc concentration <0.65 mg/L for participants <10 y independently of their fasting status and for participants >10 y, cut-offs are 0.66 mg/L for non fasting girls, 0.70 mg/L for fasting girls and non fasting boys, and 0.74 mg/L for fasting boys [26].

Iodine deficiency (IDD) was defined by a median UIC below 100 µg/L and/or a proportion of participants below 50 µg/L higher than 20%. Mild IDD was defined by a median UIC between 50 and 99 µg/l, moderate IDD by a median IUC between 20 and 49 µg/l and severe IDD by a median UIC below 20 µg/l, iodine nutrition above requirements by a median UIC between 200 and 299 µg/l, and excessive iodine nutrition by a median UIC equal or above 300 µg/l [27].

### Ethics

The protocol was approved by the ethical committee of the National Health Research of Senegal. The school directors informed parents of the selected children on the purpose and proceedings of the study. Written informed consent was obtained from all parents at the beginning of the study. Severe anemic participants received iron supplementation as treatment.

### Data Management and Statistical Analysis

Data entry, including quality checks and validation by double entry of questionnaires, was performed with EpiData version 3.1 (EpiData Association, Odense, Denmark). Data management and analyses were performed with the SAS software version 9.2 (SAS, V9.2; SAS Institute, Cary, NC).

All analyses took into account characteristics of the cluster sampling design using the appropriate survey procedures of SAS. Categorical variables were expressed as percentages and standard errors of prevalence (surveyfreq procedure). Interval variables were expressed as arithmetic means and standard errors of means (surveymeans procedure), except ferritin and transferrin whose distributions were not normal and which were also expressed as geometric means and standard errors of means.

Associations between prevalence and gender or age group were assessed by prevalence OR using logistic regression models (survey logistic procedure). Comparisons of means between gender and age groups were done through ANOVA (surveyreg procedure). Thus regression models included relevant cofounders (gender, age group or interaction according to models) to estimate adjusted ORs and differences.

## Results

In total, 604 children aged from 5–17 years participated in the study, 317 girls (52.5%) and 287 boys (47.5%) (Table 1). About half of the participants were teenagers (n = 287, 47.6%) and others were children (n = 317, 52.4%). Of the children, 26% were schooled in Dakar, 57% in nearby suburbs of Dakar (Pikine, Thiaroye, Guediawaye, Keur Massar) and 17% in far suburbs (Rufisque and surroundings). Of the children, 5.7% of the subjects had an elevated CRP and 10.6% an elevated AGP. Prevalences of incubation (high CRP and low AGP), convalescence (high CRP and high AGP), and late convalescences were (low CRP and high AGP) 1.5%, 4.2% and 6.4% respectively.

**Table 1:** Anthropometric and biochemical status of participants for all and disaggregated for children (<10 y) and teenagers (≥10 y).

	All			Children					Teenager						
	n	Mean/Prevalence	SE*	n	Mean/Prevalence		SE*	n		Mean/Prevalence		SE*		p	
BMI (kg/cm^2^)	604	15.23	0.12	287	14.51		0.14	317		15.87		0.15		<0,0001	
Thinness grade 1^a^ (%)		33.6%	2.1%		30.7%		2.8%			36.3%		2.7%		NS	
Thinness grade 2^b^ (%)		10.4%	1.2%		10.8%		1.7%			10.1%		1.4%		NS	
Thinness grade 3^c^ (%)		6.5%	0.9%		5.9%		1.5%			6.9%		1.5%		NS	
BAZ	602	**−**1.14	0.05	286	**−**1.04		0.07	316		**−**1.22		0.06		NS	
Mild thinness^d^		36.9%	2.2%		34.6%		3.2%	316		38.9%		3.0%		NS	
Moderate thinness^e^ (%) scores		12.8%	1.3%		12.2%		1.8%			13.3%		1.9%		NS	
Severe thinness^f^ (%)		5.6%	0.8%		3.8%		1.3%	316		7.3%		1.5%		NS	
HAZ	595	**−**0.13	0.06	279	0.10		0.09	316		**−**0.33		0.07		0.02	
Stunting^g^ (%)		4.9%	0.9%		3.2%		1.1%			6.3%		1.5%		NS	
Plasma retinol (µmol/l)	594	1.14	0.01	279	1.09		0.01	315		1.18		0.01		0.001	
Vitamin A deficiency^h^ (%)		3.0%	0.8%		4.3%		1.2%			1.9%		0.9%		NS	
Vitamin A marginal status^i^ (%)		35.9%	2.0%		45.5%		3.2%			27.3%		2.5%		0.00	
Plasma zinc (µmol/l)	584	0.75	0.01	269	0.75		0.02			0.75		0.01		NS	
Zinc deficiency^j^ (%)		25.9%	3.7%		23.4%		4.4%			27.9%		3.9%		NS	
Iodine (µg/l)	600	146.67	7.09	283	145.33		8.57	317		147.87		7.19		NS	
Iodine <20 µg/l (%)		1.3%	0.47%		0.7%		0.5%			1.9%		0.9%		NS	
Iodine ≥20 µg/l and <50 µg/l (%)		6.0%	1.7%		6.0%		2.2%			6.0%		1.9%		NS	
Iodine ≥50 µg/l and <100 µg/l (%)		25.5%	2.9%		26.5%		3.5%			24.6%		3.3%		NS	
Iodine ≥100 µg/l and <200 µg/l (%)		44.8%	2.8%		46.3%		3.4%			43.5%		3.4%		NS	
Iodine ≥200 µg/l and <300 µg/l (%)		17.0%	2.5%		15.2%		3.3%			18.6%		2.4%		0.07	
Iodine ≥300 µg/l		5.3%	1.3%		5.3%		1.6%			5.4%		1.6%		NS	
Hb (g/l)	596	12.58	0.05	279	12.51		0.08			12.65		0.09		NS	
Anemia^k^ (%)		14.4%	1.5%		13.3%		1.9%			15.5%		2.3%		0.07	
Ferritin (µg/l)**	594	24.86	0.64		22.81		0.64	315		25.05		0.64		NS	
Low ferritin^l^ (%)		21.4%	1.8%		23.3%		2.5%			19.7%		2.0%		NS	
Transferrin receptor (mg/l)**		8.76	0.50		7.94		0.50			7.67		0.50		NS	
High transferrin receptor^m^ (%)		33.3%	2.1%		35.5%		2.9%			31.4%		3.0%		NS	
Body iron (mg/l)		2.56	0.13		2.32		0.16			2.77		0.19		NS	
Negative body iron deficiency (%)		17.7%	1.6%		20.4%		2.4%			15.2%		1.6%		NS	
ID according to FER and sTFR(%)		39.1%	2.4%		41.2%		3.1%			37.1%		3.3%		NS	
IDA according to FER, sTFR and Hb(%)		10.6%	1.4%		10.0%		1.8%			11.1%		2.0%		0.21	
IDA according to BodyIron and Hb(%)		7.1%	1.1%		6.8%		1.4%			7.3%		1.5%		NS	

standard error;

geometric means NS non significant.

^a^ consistent with WHO adult 17≤ BMI <18.5 (IS reference);

^b^ consistent with WHO adult 16≤ BMI <17 (IS reference);

^c^ consistent with WHO adult BMI <16 (IS reference);

^d^ BAZ<−1 z-scores and ≥−2 z-scores (WHO reference);

^e^ BAZ<−2 z-scores and ≥−3 z-scores (WHO reference);

^f^ BAZ<−3 z-scores (WHO reference);

^g^ HAZ<−2 z-scores (WHO reference);

^h^ retinol<0.7 µmol/L;

^i^ retinol<1.05 and ≥0.7 µmol/L;

^j^ zinc<0.65 mg/L for children<10 y, zinc<0.70 mg/L for fasting girls>10 y and non fasting boys>10 y, zinc <0.66 mg/L for non fasting girls >10 y, zinc <0.74 µg/L for fasting boys>10 y;

^k^ hb<11.5 g/dL (<12 y), hb <12.0 g/dL (children<152 y and girls>15 y), hb<13.0 g/dL (boys>15 y);

^l^ corrected ferritin <12 µg/L;

^m^ sTFR>8.3 mg/L.

Whereas half of the mothers had a job (54%), only 26% finished primary school. Most (93%) households had electricity, but only 41% had a fridge.

Six hundred and four school children participated to the study and were measured for anthropometry, with 596 blood samples being obtained. 12 samples are missing, due to the refusal of 3 children and insufficient blood collection from 9 others. Hb was measured on all blood samples whereas CRP, AGP, FER, TFR were measured on 594 samples and Zn on only 584 due to insufficient blood volume. A total of six hundred urine samples were collected with only 4 children refusing. Due to the loss of labels during the transfer of samples, it was decided that 4 would not be measures for iodine.

Anthropometric characteristics and micronutrient status of participants are shown in Table 1, for all and disaggregated by age group. Less than 5% of the participants were stunted. Mean HAZ was significantly lower in teenagers than in children (P = 0.02) and had a tendency to be lower in boys than in girls (P = 0.08). Cumulate moderate and severe thinness measured with BAZ was much more prevalent, affecting almost 20% of participants without any statistical difference between boys and girls or age groups. Both severe thinness (BAZ <−3 z-scores) and grade 3 of thinness (equal to adult BMI <16 kg/cm^2^) were found in around 6% of children. Prevalence of moderate thinness (BAZ ≥−3 z-scores and <−2 z-scores) was 13% while prevalence of grade 2 of thinness was 10% (equal to adult BMI between 17 and 16 kg/cm^2^). Mild thinness (BAZ ≥−2 z-scores and <−1 z-scores) was 37% and slightly higher than thinness grade 1 whichwas 34% (equal to adult BMI between 16 and 18.5 kg/cm^2^). Only 3.0% of participants were overweight and two participants were obese (0.3%).

**Figure 1:**
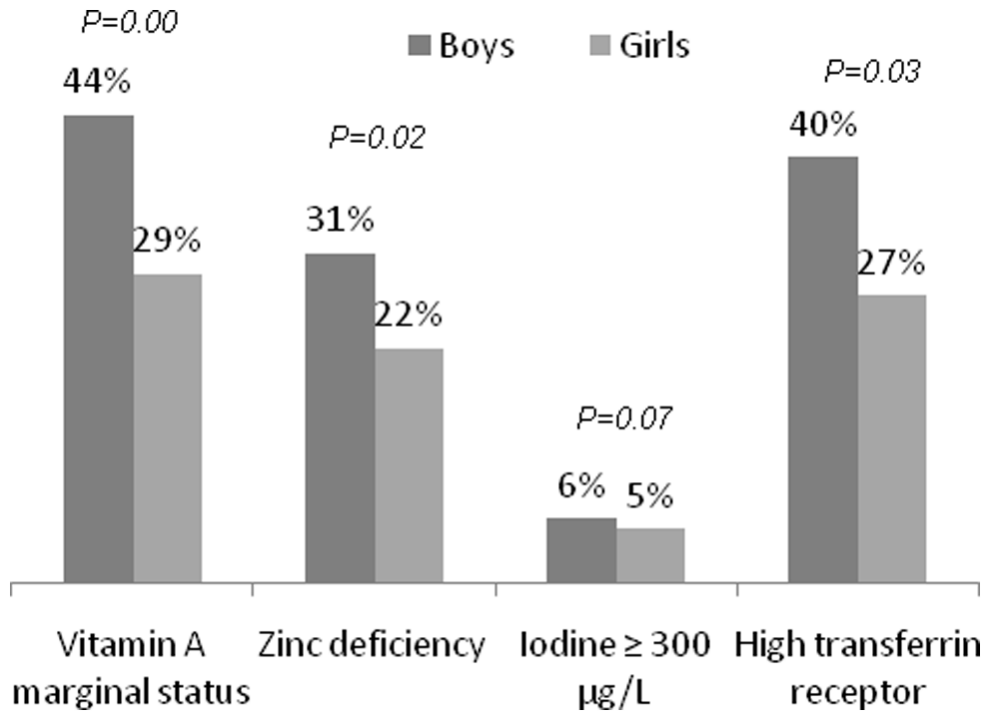
Gender-related differences in prevalence of abnormal status.

Fourteen percent of the participants were anemic. No significant difference was observed between gender groups but teenagers had a tendency to be more affected by anemia than children (P = 0.07). Only 3 participants had severe anemia. Prevalence of low FER was 21% and prevalence of high TfR was 33%. Mean TfR was significantly higher in boys than in girls (P = 0.02) as well as percentage of high TfR (P = 0.03) whereas there was no significant difference between children and teenagers. Prevalence of ID was found in approximately one third of participants, without any difference between age or gender groups. Prevalence of negative body iron content was 18%. Iron deficiency anemia was 11% when ID was defined by high TfR and/or low FER and 7% when ID was defined by negative body iron. While VAD was present in 3% of the participants, none had severe vitamin A deficiency. In contrast, approximately 40% of the participants had marginal VAD. Mean RBP was significantly lower in boys (P = 0.01) than in girls and in children compared to teenagers (P = 0.001). Moreover boys were significantly more affected by marginal VAD than girls (P = 0.003; Figure 1) and children were more affected than teenagers (P<0.0001). ZnD was highly prevalent at 26% of all participants and affected boys more than girls (P = 0.02, Figure 1). Median UIC was 137 µg/L with 7% of the participants having UIC <50 µg/L and 26% having UIC ≥50 µg/L and <100 µg/L. Only 1% had IUC <20 µg/L. Mean UIC was significantly higher in boys (P = 0.04) and the prevalence of very high UIC (>300 µg/L) tended to be higher in boys (P = 0.07, Figure 1). 22% of participants had elevated UIC, 17% between 200 and 299 µg/L and 5% above 300 µg/L. The Table 2 reports the public health significance of each nutritional disorder reported in the current paper according to international references [27]–[32].

**Table 2:** Public health significance of nutritional disorders in school-aged children from Dakar.

	Indicators prevalence	Public health significance
Vitamin A deficiency	3.0%	mild
Zinc deficiency	25.9%	mild
Iodine deficiency disorders	UI <50 µg/L 7%	No
	median UIC 136.8 µg/L	
Anemia	14.4%	mild
Iron deficiency	lowFer 21.4%	yes
	highTfR 33.3%	
Thinness	50.6% (corresponding to BMI<18.5 kg/cm^2^in adults)	High prevalence (serious situation)
Stunting	prevalence 4.9%	Low prevalence

No association was found between micronutrient deficiency and thinness/stunting. Multiple micronutrient deficiency was not prevalent. No association has been found between iron deficiency, zinc deficiency, thinness, stunting, and socioeconomic characteristics (education level of the mother, socioeconomic status of the mother and the household head).

## Discussion

The present study, which was carried out in a representative sample of school children attending state primary schools in Dakar, showed multiple nutritional problems. Prevalence of thinness, anemia and deficiencies of iron and zinc were high. In contrast, overweight and vitamin A deficiency were less prevalent. More than 50% of the children had evidence for inadequate (too low or too high) iodine intakes.

In the present study, both references from WHO and IS have been used because they are relatively new and under-used: in 376 previous studies on school-children, neither references were used [6]. The comparison between the 2 references shows only slight differences. Prevalence of grade 3 thinness as defined by Cole was similar to severe thinness defined by WHO, which is consistent with a recent study carried out in Seychelles [33]. Prevalence of grade 2 and 1 thinness (Cole) were slightly lower than moderate and mild thinness respectively as defined by WHO. As reported earlier, the WHO reference gives higher prevalence of especially mild thinness, regardless of age of the subjects.

Many factors underline the high prevalence of thinness observed in the present study. For instance, infections or communicable diseases could have contributed to thinness. In our study, 12% of children had signs of inflammation as indicated by the elevated concentrations of acute phase proteins. Malaria, estimated to affect 8% of children, or diarrhea, are major causes of disease in Senegal. Other infections such as HIV/AIDS or tuberculosis are less prevalent in Senegal, with 0.7% of adults and 0.4% of people aged 15–24 y being HIV-positive [34] and 0.2% of adult population is affected by tuberculosis [35]. One might therefore assume that thinness is closely related to low dietary intake. It has been demonstrated that whereas malnutrition in infancy and young childhood is strongly related to stunting, thinness is an indicator of malnutrition in all age groups and suggest recent undernutrition [36]. The low prevalence of stunting (5%) would indicate that nutritional intake in the first years of life was probably adequate, with under nutrition only appearing later in childhood.

A study on food practices and nutrition in urban regions of Senegal identified several reasons for food insecurity and undernutrition inurban school-aged children [12]. These are mainly related to changing dietary habits. For instance, urban populations tend to decrease domestic foods in favor of street foods, with only one meal being home-cooked and consumed in the afternoon. For breakfast and dinner, families tend to increasingly buy snacks in the street. Worryingly, children attending urban state schools do not have lunch at home, while simultaneously not having access to canteens and thus school meals. During school days, they buy cheap foods from street sellers, which most often lack in proteins and micronutrients. This is highlighted by the finding that in the present study that found almost 6% of the participants were categorized as severe malnourished (BAZ <−3 while only 1.9% of children under 5 y living in urban areas of Senegal have weight for height <−3 z-score [3].

Advocacy and more importantly actions for intervention improving the nutritional status of school children in Senegal are thus vital. Offering affordable and healthy meals in school canteens is but only one option available.

The prevalence of anemia (14%) still represents a public health problem [29]. This prevalence was less than half of the prevalence of anemia reported in school children in Dakar 18 years ago (35%) [11]. Another study carried out in Dakar in 2003 on a small sample of seven years old children indicated a prevalence of anemia of 39% [37]. These combined data suggest that anemia has decreased among Senegalese school children in Dakar over the last decade. Compared to other African countries, the prevalence of anemia found in the present study was much lower than the prevalence of ∼40% recently found in 2 studies among school children in Burkina Faso and Cote d’Ivoire [38]. In contrast, a recent study carried out in 6–16 y old school children in rural Kenitra, Morocco found a similar anemia prevalence of 12% [39].

Prevalence of ID defined either by low ferritin and/or by high transferring receptor concentrations was high (39%). However, in this study more participants had elevated TfR (33%) than low ferritin (21%) or negative body iron (18%). Both indicators measure different stages of iron deficiency, with low ferritin indicating the depletion of iron stores and elevated TfR related to the iron-deficient erythropoiesis, indicating a later stage of iron deficiency [38]. To our knowledge, it is the first time that ID in a population was more related to abnormal TfR values than to low ferritin. However, a study carried out in 5–15 y old children in Cote d’Ivoire recommends the use of higher cut-offs for TfR i.e. 9.4 mg/L for African populations (versus 8.3 mg/L used in our study) to improve its performance in defining iron status in children. However, the authors concluded that TfR had only a modest sensitivity and specificity in identifying iron deficiency, regardless of the diagnostic cutoffs chosen [38]. When applying this cut-off, the prevalence of high TfR decreased from 33 to 20%, which was very close to the 18% of children with a negative total body iron. This study suggests that a cut-off of 9.4 mg/L for TfR might be better in these settings. Even with this higher cut-off, the prevalence of ID defined by either abnormal ferritin or TfR remained high at 29%.

ID prevalence was more than double the prevalence of anemia demonstrating that anemia prevalence rates cannot be used as a proxy indicator for ID [21]. To allow correction of ferritin concentrations in the presence of inflammation, iron status indicators have to be included in epidemiologic studies combined with indicators of inflammation. In addition, the prevalence of iron deficiency anemia (IDA) was low and only represented half of the anemia prevalence, indicating that other factors contributed to anemia. Malaria infection and hemoglobinopathies such as sickle cell are likely contributors with prevalences of 8% and 10% respectively [40], [41]. The prevalence of vitamin A deficiency, which can contribute to anemia, was low. However, other micronutrients such as vitamins B12 and folic acid were not measured. These deficiencies could have played a role in the etiology of the non-ID anemia found in the present study [42].

In Burkina Faso, 40% of school children aged 7–14 y in Ouagadougou are vitamin A deficient [43], whereas 59% of children aged 6–9 y are vitamin A deficient and 8% have severe VAD in rural Northern Ethiopia [44]. Both studies used retinol concentrations as an indicator while the current study uses RBP concentrations. However, at low plasma retinol concentrations, RBP concentrations is a less sensitive indicator of vitamin A status, as more unbound RBP appears in the circulation [45], [46]. Using RBP concentrations may therefore have underestimated the prevalence of vitamin A deficiency in the current study. Mean RBP concentration was significantly lower and prevalence of marginal vitamin A status higher in boys and in children. Although the study notes the differences in vitamin A deficiencies between boys and girls, with higher risks for boys, it does not provide a clear answer for this variance. However, similar sex differences for other micronutrients such as iron and zinc have been reported before [47].

Zinc deficiency (ZnD) was highly prevalent with almost ¼ of all school children affected representing a significant higher rate than the cut-off of 20% indicating a public health problem [30]. Furthermore, in consensus with similar studies, boys were significantly more affected than girls. The higher requirements of zinc for boys generally suggest that boys are more sensitive to ZnD [48]–[51] [48]–[51]. Indeed, boys have higher proportion of muscles per kilogram body weight, which contains a higher content of zinc than fat, and the growth rate of boys is higher than girls [26].

According to both the median UIC (136.8 µg/L) and proportion of children with UI<50 µg/L (7%), iodine deficiency is not a major public health issue in school children in Dakar. Iodine nutrition in Dakar is supposed to be good, as Senegal introduced iodized salt in 1995. According to a national survey in 2006, 70.5% of households consume adequate iodized salt (>15 ppm) [3]. Moreover, Dakar’s geographical location near the sea ensures adequate intake from seafood [3]. Nonetheless, 33% of children had insufficient iodine intake (UIC below 100 µg/L), and although this is lower than WHO estimates for Africa in general (41% of children 6–12 years having UIC<100 µg/L [52]), it is still a considerable percentage of children. Worrying, only 45% of the children had adequate iodine intake (UIC between 100 and 199 µg/L), with 22% of the children being at risk for iodine-induced hyperthyroidism (UIC above 200 µg/L) and 5% of children risk adverse health consequences (UIC above 300 µg/L) like iodine-induced hyperthyroidism or autoimmune thyroid disease) [27] [27]. Two studies in non coastal countries Lesotho and Angola on children from rural schools reported respectively 94% and 78% of children with UIC<50 µg/L and only 0% and 2% children with UIC≥200 µg/L. As iodine deficient populations seem to be more sensitive to health consequences of excessive iodine intakes [52], urgent attention is needed for iodine nutrition in school children in Dakar.

Apart from gender and age related differences, it was not possible to identify a specific vulnerable socioeconomic group of children, or a group of children more affected by malnutrition. Nutritional issues seem to randomly affect the whole population of the study.

Not including non-school attendees is a limitation of the study, as they might be the most vulnerable to malnutrition. However, one of the objectives of the study was to provide information to the education ministry in order to illustrate nutritional disorders in urban school attendees. The goal was to reorient the strategy of school-feeding programs towards broader coverage, as only rural state schools received school meals. The entire population of urban school-aged children and teenagers should be taken into account by nutrition policy.

## Conclusion

To conclude, many school-aged children in urban Senegal have a poor nutritional status, as the high prevalence of iron and zinc deficiency illustrate. Furthermore, iodine intake as indicated by urinary iodine concentrations was either too low or too high in over half of the children. Although the low rate of stunting in the population suggests adequate nutrition during the first years of life, the prevalence of thinness going up to almost 20% remains alarming. The study highlighted that the transition from home meals in the pre-school period to self-catering at school is most likely the basis for these multiplex of nutritional problems. The study therefore stresses the need for nutritional interventions to improve dietary quality and quantity of school children in Senegal.
